# Monitoring Attention in ADHD with an Easy-to-Use Electrophysiological Index

**DOI:** 10.3389/fnhum.2018.00032

**Published:** 2018-02-01

**Authors:** Goded Shahaf, Uri Nitzan, Galit Erez, Shlomo Mendelovic, Yuval Bloch

**Affiliations:** ^1^BrainMARC LTD, Yokneam, Israel; ^2^Shalvata Mental Health Center, Hod HaSharon, Israel; ^3^Sackler Faculty of Medicine, Tel Aviv University, Tel Aviv, Israel; ^4^Beer Yaakov Mental Health Center, Beer Yaakov, Israel

**Keywords:** ADHD, EEG, CPT, brain engagement index, methylphenidate

## Abstract

Attention deficit hyperactivity disorder (ADHD) involves characteristic electroencephalographic (EEG) activity. We developed a single-channel EEG marker for attention: the Brain Engagement Index (BEI’). In this study, we evaluated the use of BEI’ for distinguishing between ADHD patients and controls, and for monitoring the effect of pharmacological treatment on ADHD patients. The BEI’ values of 20 ADHD patients and 10 controls were measured using a 1-min auditory oddball paradigm and a continuous performance test (CPT) task. We showed that CPT BEI’ is trait-specific and separates controls from ADHD patients. At the same time, oddball BEI’ is state-specific and identifies differences in attention level within the two groups of ADHD participants and controls. The oddball BEI’ also associates with response to treatment, after distinguishing between treatment effect and learning/time effect. The combined use of this marker with common computerized tests holds promise for research and clinical use in ADHD. Further work is required to confirm the results of the present study.

## Introduction

To date, the diagnosis of Attention deficit hyperactivity disorder (ADHD) has been based on clinical evaluation and questionnaires. Although objective computerized tests of attention, such as continuous performance test (CPT), are used at times in clinical practice, their specificity and sensitivity, such as the CPT, has not justified recommending its use in practice guidelines. Due to the subjective nature of ADHD diagnosis, it seems important to develop biomarkers that reflect pathological understanding and are practical for use (Rosenberg et al., 2016).

ADHD involves characteristic electroencephalographic (EEG) activity (Loo and Barkley, 2005). Electrophysiological markers that differentiate between ADHD patients and controls are based on three key analysis paradigms: (i) raw EEG analysis methods and tools (Arns et al., 2013), such as the NEBA LTD commercial tool evaluation based on the theta/beta ratio (Snyder et al., 2015; Gloss et al., 2016), which was approved by the Food and Drug Administration (FDA); (ii) event-related analysis methods, such as event-related potentials (ERP) and event-related synchronization/desynchronization (ERS/ERD; Fisher et al., 2011; Shahaf et al., 2015); and (iii) task-related analysis, which involves raw EEG analysis methods applied to samples collected during designated tasks, for example, CPT (Loo et al., 2009). Tasks such as CPT, often used in ERP analysis (Sunohara et al., 1999), are analyzed in a task-related manner, without synchronization to stimulus time. The electrophysiological markers for attention tend to normalize with effective treatment (Verbaten et al., 1994; Sunohara et al., 1999).

In the last several years, we developed an effective single-channel marker for attention (Shahaf et al., 2015, 2017; Bartur et al., 2017). Our work shows that attention processes can be efficiently monitored over a wide frontocentral area with the use of prefrontal electrodes, in a manner that distinguishes between patients with ADHD and controls (Shahaf and Pratt, 2013; Shahaf et al., 2015). We found it effective to use a simple and minimal setup of two electrodes for monitoring prefrontal activity. We also simplified the EEG analysis, to adjust the extraction of relevant attention-related markers from a short sample, on the scale of 1 min, based on template matching (Vijayalakshmi and Abhishek, 2010; Bartur et al., 2017; Shahaf et al., 2017) of the marker identified in the averaged ERP (Shahaf and Pratt, 2013; Shahaf et al., 2015). Template matching involves the search in the sampled EEG data for an *a priori* given pattern. We follow in this regard a known methodology that scans the raw EEG data for patterns identified in the averaged ERP signal (Jaśkowski and Verleger, 1999).

Note that while in the averaged ERP sample our marker is time-locked to the stimulus (Shahaf and Pratt, 2013; Shahaf et al., 2015), we found that the marker onset is much more variable at the single-trial level. According to the literature, such a large temporal variability, on the scale of many hundreds of milliseconds, is larger for low frequency EEG activity and has been associated with the amplitude and phase of pre-stimulus oscillations (Stefanics et al., 2010). Because of this large variability in evoked response latency, in our single-trial studies, we did not time-lock the template matching at the single-trial level (Shahaf et al., 2017). We followed this line of thinking, relating the sampled activity to the superposition of attention-related processes and preceding oscillations to the continuous EEG analysis. We showed that even without known timed external events, it is possible to evaluate the level of attention from short samples of continuous EEG on the basis of the prevalence of such template matches of attention-related activity (Bartur et al., 2017). In this work, we follow the same template matching approaches, with and without external events.

Extracting a relevant marker for attention from a simple-to-use EEG system may have significant practical value. It may be used conveniently in any real-life clinical setting during a CPT session to improve diagnostic precision (Loo et al., 2009). An easy-to-acquire 1-min sample can be used in home settings on a regular basis, with the potential to provide regular monitoring, which can be valuable for treatment titration (Vitiello et al., 2001).

In the present study, we evaluated the ability of our electrophysiological marker, the Brain Engagement Index (BEI’), a marker for sustained attention (Bartur et al., 2017; Shahaf et al., 2017), to distinguish between ADHD patients and controls. We also evaluated the effect of pharmacological treatment on the BEI’. Our main hypothesis was that it is possible to differentiate between ADHD patients and controls using the BEI’. We measured the BEI’ using a 1-min auditory oddball paradigm and during a CPT task.

## Materials and Methods

### Participants

Twenty young adults diagnosed with ADHD (15:5 males:females, 29.05 ± 6.12 years old (mean ± standard deviation)), and 10 age-matched controls (3:7 males:females, 29.3 ± 5.48 years old (mean ± standard deviation)) were included in the study. Participants were recruited by advertisements in relevant ADHD and student Internet forums. Candidates underwent a psychiatric evaluation, using a semi-structured interview based on DSM5 criteria, at the Shalvata Mental Health Center, in Hod Hasharon, Israel, to establish the clinical diagnosis of ADHD. Exclusion criteria included: (i) diagnosis of any active psychiatric disorder or diagnosis of bipolar disease, psychotic disease, or major neurological disorder; (ii) recent history of substance or alcohol abuse; (iii) major cognitive impairments; and (iv) significant hearing impairment. The control group underwent a similar evaluation, and a diagnosis or a history of a diagnosis of ADHD was added to the above-mentioned exclusion criteria. All participants signed an informed consent form. The study was approved by the local ethics committee of Shalvata Mental Health Center (NIH clinical trial identifier: NCT02625805). Each participant was presented with the study procedure and was only included in the study after signing an informed consent.

### Tools

#### Questionnaire

All participants completed the Adult ADHD Self-Report Scale (ASRS; Kessler et al., 2005).

#### Computerized Assessment

We used a computerized CPT, the MOXO CPT. MOXO is comprised of eight blocks, each lasting about 140 s. The blocks present target and no-target stimuli. They differ in the types of additional distracting stimuli used: none, visual, auditory, or combined. Each type of distracting stimulus is used in two of the eight blocks (using no distracting stimuli in the first and last block). MOXO reports four indices for each block, and for the entire test: attention, timing, impulsiveness and hyperactivity (Cassuto et al., 2013).

#### Electrophysiological Tool

The electrophysiological data were recorded from the NeuroSky EEG MindWave single-channel system (NeuroSky Inc., San Jose, CA, USA, CE-authorized), with one frontal electrode (~Fpz) and one reference electrode on the earlobe, using a sampling rate of 512 Hz. In previous works, we noted that our template marker could be extracted from any sagittal or para-sagittal electrode in the central and frontal regions, if the reference is peri-auricular (Shahaf et al., 2015). We chose the Fpz frontal location and the earlobe reference location because a setup of dry electrodes, which sample below the hairline, is easier to use. The MindWave EEG headset uses dry EEG electrodes (Rebolledo-Mendez et al., 2009). The sampled data were transferred through a wireless connection to the experiment computer for offline processing. Each sampling session involved 5 min of stimulus-free recording and 5 min of recording during the execution of the auditory oddball protocol. The oddball stimuli consisted of 1000 and 2000 Hz tones of 40 ms duration, presented binaurally at ~60 dB. The stimuli were comprised of a frequent tone (1000 Hz) presented 80% of the time, and a rare tone (2000 Hz) presented 20% of the time. Inter-stimulus interval was selected randomly in the range of 2–3 s. Only data from the first minute of the stimulus-related samples were used in the analysis. Note that we did not use the oddball paradigm for event-related analysis, but rather for task-related analysis. The role of the paradigm was to maintain a higher level of attention, as described in the literature on task-related analysis studies (Loo et al., 2009). Therefore, we did not differentiate between the two types of stimuli in the data analysis.

### Procedure

After completing the informed consent process and filling out the questionnaires, each candidate underwent a full clinical evaluation by an experienced psychiatrist, including a semi-structured portion, to verify (or exclude, for the controls) that participant meet DSM 5 criteria for ADHD (*DSM 5*. 2013. American Psychiatric Association). ADHD participants who used regular pharmacological treatment (mostly methylphenidate immediate release, 10–20 mg, or slow release, 20–60 mg) were instructed to use the same treatment during the study. Participants who did not use a regular treatment were prescribed by the study psychiatrist a standard pharmacological treatment. We verified that no treatment was taken on the study day, before starting the sampling.

**Figure 1:**
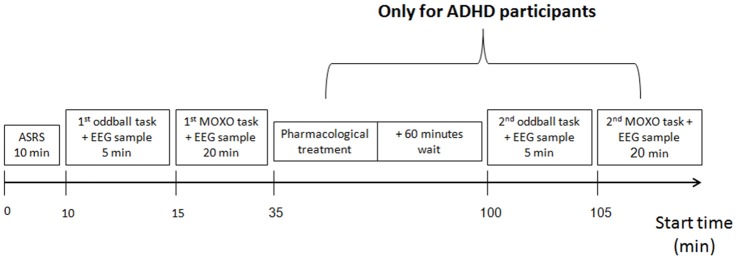
Experiment procedure. All participants underwent ADHD self-report scale (ASRS) evaluation, then electrophysiological measurement, using the auditory oddball and the MOXO continuous performance test (CPT). Next, attention deficit hyperactivity disorder (ADHD) participants received pharmacological treatment, and after a 60-min wait were re-measured, using a second auditory oddball and a second MOXO CPT.

Each participant was measured by the MindWave system for 5 min, then using the auditory oddball protocol, for another 5 min. Next, participants took the MOXO CPT test, while continuing to be measured by the MindWave system. ADHD participants were then treated pharmacologically either with their standard treatment or with the treatment prescribed to them by the study psychiatrist. After an interval of 1 h, the ADHD participants were re-measured with the MindWave system for 5 min before and for 5 min during a second auditory oddball task, after which they took a second MOXO CPT test. The experimental flow is presented in Figure 1. All auditory oddball measures were conducted with eyes closed to reduce a possible electrophysiological artifact of eye movements.

### Data Analysis

#### Computerized Cognitive Data Analysis

##### The normal, intermediate and low CPT groups

Each MOXO session produces four indices: attention, timing, impulsiveness and hyperactivity. Each index is divided into three levels, which are color-coded in the test output (Cassuto et al., 2013). The low performance level, which is more than 2 standard deviations below the normal population mean (red), the intermediate performance level, which is between 1–2 standard deviations below the normal population mean (yellow), and the normal performance level, which 1 standard deviation below the normal population mean or above (dark and light green). Based on their worst performance on any of the measures, participants were divided into normal, intermediate and low performance. Participants received a normal global performance index, if all their four indices (attention, timing, impulsiveness and hyperactivity) were at the normal levels; and a low global performance index, if at least one of the four indices was at the low performance level. In all other cases, participants received an intermediate global performance index (because their worst index out of the four was intermediate).

##### The standard+ and standard− groups

We further grouped participants based on their global performance index in the following manner: ADHD participants with normal global performance indices were included in the ADHD standard+ group; ADHD participants with intermediate or low global performance indices were included in the ADHD standard− group. Similarly, controls with normal global performance indices were included in the standard+ control group, and those with intermediate or low global performance indices were included in the standard− control group.

##### The treatment effect vs. time/learning effect groups

The first and last blocks of each MOXO session do not involve distracting stimuli. Each of the MOXO indices (attention, timing, impulsivity, and hyperactivity) is graded in each block on a scale ranging 0–100. We averaged the four indices for the first and last blocks, generating a block grade for these two basic blocks, without distracting stimuli. For ADHD participants, we computed the within-session difference, which is the difference in between the first and last blocks in the first MOXO session, which do not involve distractors. We also computed the difference between the first basic block index of the second MOXO session (after pharmacological intervention) and the best basic block index in the first MOXO session: the between-sessions difference. We allocated ADHD participants to the treatment effect group if the between-sessions difference was greater than the within-session difference; and we allocated ADHD participants to the time/learning effect group if the within-session difference was greater than the between-sessions difference.

#### BEI’ Analysis

**Figure 2:**
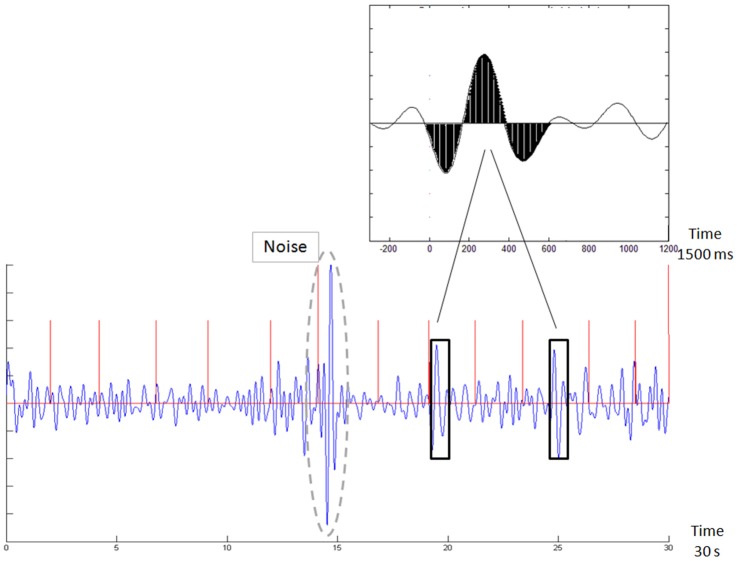
Demonstration of template matching. The template is emphasized in black in the top inset. The new sample in the bottom chart is scanned with a moving window, following normalization to the [−1,1] range. Whenever a match is found (black rectangles), it is counted. Two such outstanding matches are emphasized by black rectangles. The brain engagement index (BEI) is a normalization of this count to the [0,1] range. The bottom chart also shows an automatically rejected noisy sample (surrounded by a dashed gray line). The red vertical lines denote the stimulus times.

BEI is a standard computation we use for analyzing the EEG data obtained from the first minute of the stimulus-related sample. The computation is based on template matching (Vijayalakshmi and Abhishek, 2010). This technique uses a basic template, which is compared with the sampled signal. In this case, the template consisted of a 1500 ms, attention-related, averaged ERP delta bandpass activity (Shahaf et al., 2015), which was matched with a moving window of the same size in the sampled signal. The matching was done as follows: (i) the 1-min sample was divided into segments of 10 s; (ii) each segment was filtered in the delta bandpass [1–4 Hz]; (iii) the data points in the filtered segment were normalized to the [−1,+1] range, where −1 denotes the most negative deflection within the filtered segment and +1 the most positive deflection; (iv) the process of filtering and normalization to [−1,+1] was also performed for the 1500 averaged delta ERP wave shown in Figure 2, top inset, to generate the template (derived from Shahaf et al., 2015); (v) the normalized sampled segment was scanned by a moving window of 1500 ms, in 1 ms moving steps; (vi) the averaged distance between the moving window data and both the template and the template opposite (negation of template) was computed (Figure 2); (vii) if the averaged distance to either the template or the template opposite was less than a threshold of 0.5, the match count was increased, provided that no other matching was found in a previous window, partly overlapping the current one; (viii) if the averaged distance was more than the threshold, the no-match count was increased, provided that no other no-matching was found in a previous overlapping window; (ix) the BEI is the division of the match count by the no-match count (maximum BEI value is set to +1, therefore BEI is represented on a scale of [0, 1]); (x) for every 1500 ms window, we also computed the standard deviation mean ratio. We used manual inspection to determine whether this ratio is greater than 1. If yes, the sampling is likely to be noisy and the 1500 ms samples were rejected (not included in the computation). If more than 1 non-overlapping 1500 ms windows were rejected within a given 10-s segment, the entire segment was automatically rejected. At least 3 10-s segments were required to be valid to generate a valid BEI for the entire sample; otherwise, the entire sample was rejected as noisy and was not included in the next stages of analysis. The BEI’ is computed as the average distance of the 10-s BEI values from 0.7, which was found to be the average BEI level during recruiting tasks (Bartur et al., 2017; Shahaf et al., 2017).

Because of the large single-trial variability (Pfurtscheller, 2001), template matching was not time-locked with the stimuli. No distinction was made between the two types of stimuli.

### Statistical Analysis

We used independent *t*-tests to compare the BEI’ of ADHD patients and controls. We evaluated the comparison of BEI’ for both ADHD patients and controls, distinguishing between participants in the standard+ and the standard− groups, using a two-way ANOVA. We evaluated the comparison of BEI’ change percentage for ADHD patients from the first to the second test, distinguishing between the treatment effect and the time/learning effect groups, using a chi-square test. We used a basic alpha level of 0.05 for all statistical tests. We evaluated both the oddball BEI’ and the MOXO BEI’ for the comparison of BEI’ between ADHD patients and controls, as well as for the comparison of BEI’ in ADHD patients and controls, distinguishing between participants in the standard+ and standard− groups. In these dual task analyses we also performed a Bonferroni correction, using an alpha level of 0.05/2 = 0.025. Statistical analyses were performed using SPSS procedures.

## Results

### Results Obtained Using Common Tools for Diagnosing ADHD: ASRS and CPT (MOXO Test)

**Figure 3:**
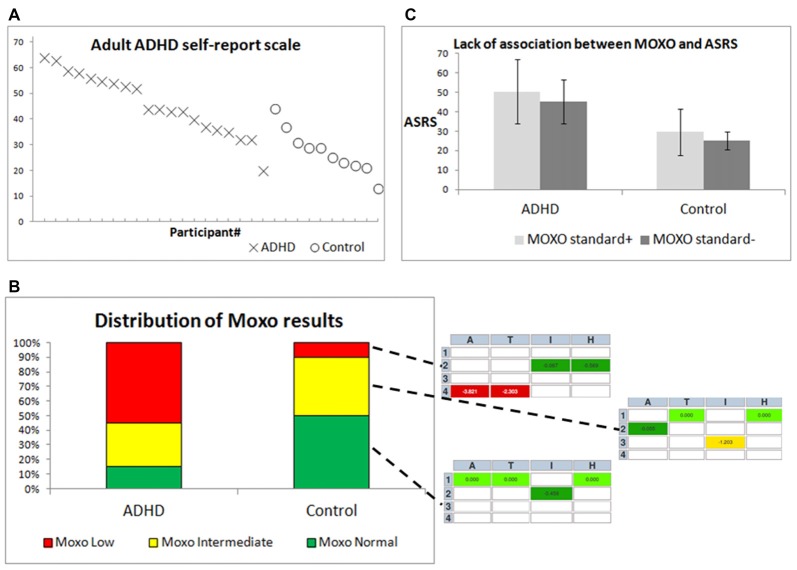
Differentiation between ADHD patients and controls by behavioral diagnostic tools. **(A)** ASRS scores for individual participants, ordered by group and by ASRS score. X = ADHD patients, O = controls. **(B)** The right inset from three MOXO reports includes four indices: attention [A], timing [T], impulsiveness [I] and hyperactivity [H]. Each index is color-coded in the report: green indicates standard or above-standard performance, yellow indicates intermediate performance and red indicates difficulty in performance. **(C)** Means ± standard deviations of ASRS scores for both groups, with distinction between MOXO standard+ and MOXO standard− participants.

As expected based on previous studies, both the ASRS (Figure 3A) and the MOXO (Figure 3B) were associated with a diagnosis of ADHD (Kessler et al., 2005; Cassuto et al., 2013). Control participants received a lower grade (mean: 27.4, standard deviation: 8.75) than did ADHD patients (mean: 46.0, standard deviation: 11.89) in the ASRS questionnaire (*t*-test, *p* < 0.001). In the MOXO test (Figure 3B), more ADHD patients (11/20) were in the low range, and more controls were in the normal range (5/10). Nevertheless, there was a large overlap in the intermediate range (6/20 ADHD patients and 4/10 controls), and in the outliers, i.e., 3/20 ADHD patients who showed normal CPT performance, and 1/10 controls who showed low CPT performance. The inset in Figure 3B demonstrates how participants are graded as low, intermediate, or normal according to their worst MOXO grade of attention [A], timing [T], impulsiveness [I] and hyperactivity [H]. There was no significant association between CPT performance (standard+ vs. standard−, Figure 3C), and the ASRS score within the two groups of participants (ADHD patients and controls; *F*_(1,29)_ ≈ 0.51; *p* ≈ 0.48).

### Comparison of BEI’ between ADHDs and Controls during Pre-treatment MOXO and First Auditory Oddball

**Figure 4:**
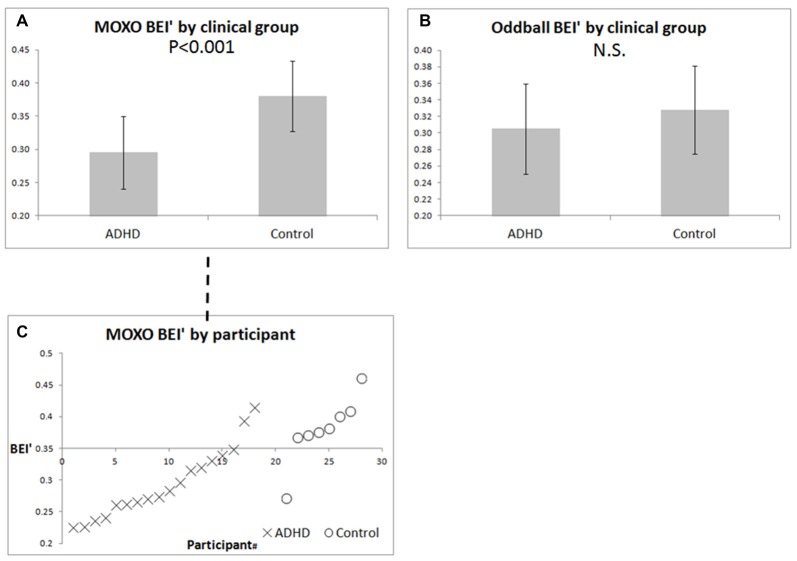
BEI’ of ADHD patients and controls during pre-treatment MOXO and auditory oddball. **(A)** Mean ± standard deviation of BEI’ during the pre-treatment MOXO test in ADHD patients and controls. **(B)** Mean ± standard deviation of BEI’ during the pre-treatment 1-min auditory oddball. **(C)** BEI’ during the pre-treatment MOXO by group and participant, ordered by BEI’ within each group. X = ADHD patients, O = controls.

For the MOXO CPT, the BEI’ differed between ADHD and controls (Figure 4A, *p* < 0.001). For the auditory oddball, the BEI’ did not differentiate between the two groups (Figure 4B, n.s.). Figure 4C details the MOXO BEI’ values of all participants whose samples were not rejected as noisy.

### Comparison of BEI’ between Participants Based on Functional Results in Pre-treatment MOXO

**Figure 5:**
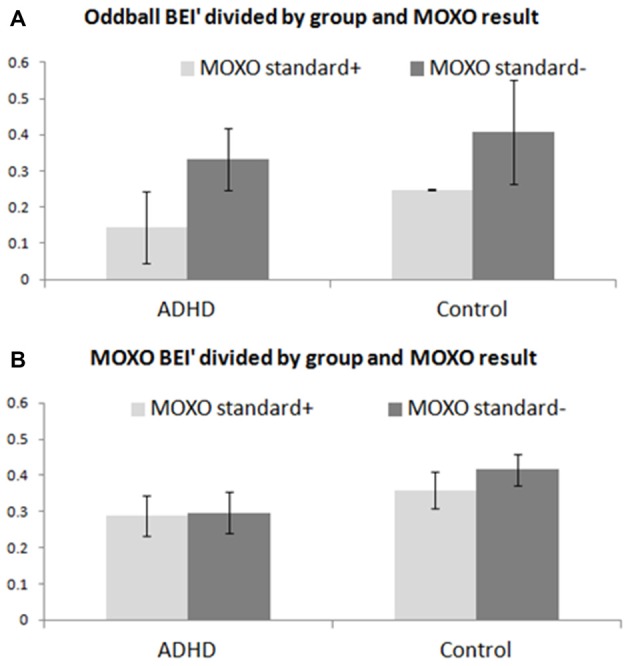
Comparison of BEI’ between participants based on pre-treatment MOXO. BEI’ comparison between participants with standard+ pre-treatment MOXO functional results and those with standard− pre-treatment MOXO functional results, in both ADHD and control groups. **(A)** Mean ± standard deviation of BEI’ during the pre-treatment 1-min auditory oddball for standard+ and standard− participants. **(B)** Mean ± standard deviation of BEI’ during the pre-treatment MOXO test for standard+ and standard− participants.

The oddball BEI’ was associated significantly with performance on the MOXO CPT for both ADHD patients and controls (Figure 5A), as opposed to lack of such association with the MOXO BEI’ (Figure 5B). A two-way ANOVA computed for BEI’ during the pre-treatment auditory oddball was found to be significant for MOXO functional results: standard+ vs. standard− within both ADHD and control groups (*F*_(1,22)_ ≈ 12.22; *p* ≈ 0.002).

### BEI’ Change (for MOXO and Auditory Oddball) between First and Second Tests, and Association with Treatment Effect

**Figure 6:**
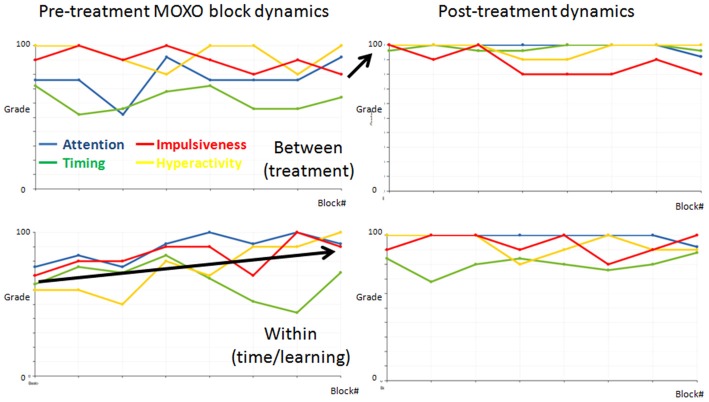
Demonstrations of functional dynamics between the MOXOs and within the first MOXO. The dynamics of the four indices (attention = blue, timing = green, impulsiveness = red, and hyperactivity = yellow) between the eight blocks in the two MOXO sessions of two representative ADHD participants. The first and last blocks in each test are similar and are comprised of basic stimuli without distractors. Each participant’s dynamics is presented in a separate row. The left graphs present the pre-treatment, the right graphs the post-treatment MOXO session. Note that for the top participant the change occurred mainly between sessions. This is a representative participant of the treatment effect group. By contrast, for the bottom participant the change occurred mainly in the first pre-treatment session. This is a representative participant of the time/learning effect group.

One of the complexities in evaluating treatment effect in cognitive testing has to do with the time/practice effect; therefore, we divided the ADHD participants into three main groups: participants for whom the change occurred mainly between the sessions and could be ascribed to treatment effect; participants for whom the change occurred mainly within the first session and could be ascribed to the time/learning effect; and participants for whom no significant change (change ≤ 5%) occurred (examples of treatment effect and time/learning effect are presented in Figure 6).

**Figure 7:**
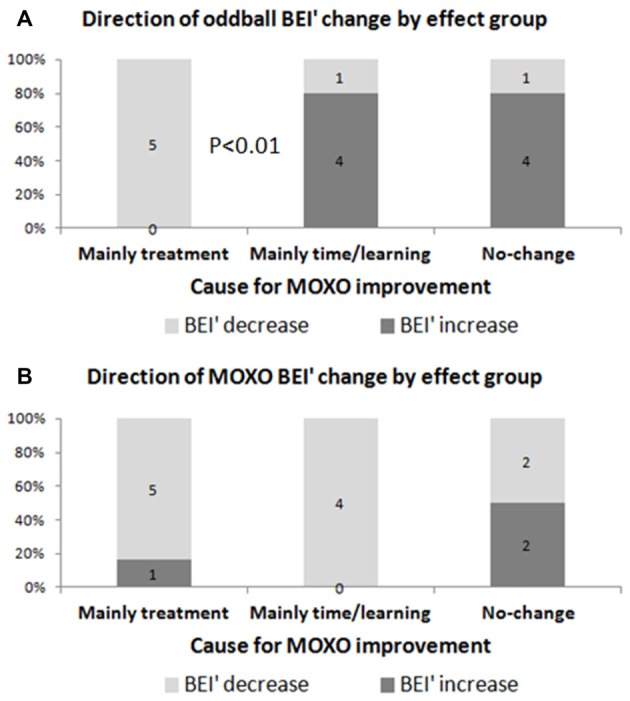
Direction of BEI’ change as a function of main contribution to improvement in MOXO. Evaluation of BEI’ change according to the main contribution to improvement in MOXO: treatment (between MOXOs) vs. time/learning (within the first MOXO). **(A)** Oddball BEI’ in ADHD patients who were affected by treatment decreased (i.e., improved) after treatment, as opposed to participants who were affected by time/learning. **(B)** MOXO BEI’ showed similar decrease for both groups.

An increase in oddball BEI’ was specific to those who had no change in the CPT or those whose change was related to time/learning, whereas a decrease in oddball BEI’ was specific to patients with CPT improvement between sessions (Figure 7). Thus, the direction of oddball BEI’ change was opposite for treatment effect and time/learning effect participants (chi-square, *p* < 0.01). The MOXO BEI’ did not show such discriminative value (n.s.). Note that a decrease in BEI’ means higher attention.

## Discussion

In the present study we conducted a preliminary evaluation of the usability of a new marker for attention in ADHD: the BEI’. The marker is practical and easy to use. The present study supports its possible use in clinical and research work with ADHD.

### Distinction between ADHD Patients and Controls

Addition of the BEI’ measurement during the performance of a cognitive task (MOXO CPT in the present study) can help identify ADHD patients. ADHD patients and controls differed in the BEI’ of the pre-treatment MOXO: average BEI’ was lower in the ADHD group. This may be unexpected, because the BEI’ (the measure of the distance from 0.7, representing a high level of attention) is expected to be lower when there is better recruitment of attention. But other electrophysiological analyses of task-related samples also reported higher attention-related indices in the ADHD group (Loo et al., 2009). Such findings are consistent with current theories suggesting that the major dysfunction in ADHD is in working memory (Holmes et al., 2014). ADHD patients may need greater attention recruitment to perform tasks, such as CPT, which for controls are significantly easier, and therefore require the recruiting of less attention.

MOXO BEI’ can help distinguish ADHD patients from controls (Figures 3A,B). The contribution of electrophysiological markers to behavioral ones is not a novelty (Shahaf et al., 2015). The novelty in the present work is the suggestion that it may be possible to achieve this distinction with a simple setup. The electrophysiological distinction between ADHD patients and controls may be used to facilitate the necessary improvement in CPT performance (Preston et al., 2005). If individuals perform poorly on the CPT test but their BEI’ is high, it may indicate reduced effort rather than ADHD. If individuals perform well in the CPT test, but their BEI’ is low, it may indicate compensation for the difficulty rather than absence of ADHD.

### Basic Attention State Captured by the Oddball BEI’

Participants whose pre-treatment MOXO function was standard+, differed in their auditory oddball BEI’ from those whose pre-treatment MOXO function was standard−, in both ADHD and control groups. We suggested above that ADHD patients require greater recruitment of attention during the MOXO task. Nevertheless, within both groups there is still a certain basic level of attention that is required for effective performance on the CPT, and the question arises whether each participant can recruit at least such a level of attention. The auditory oddball condition, which does not involve any active response by the participant, may be viewed as less attention-recruiting, and as such it may be an adequate tool for evaluating the current basic attention level of the participant. Thus, the oddball BEI’ may correlate with attention state, whereas the MOXO BEI’ may correlate with attention trait. Because of the intra-group association between the oddball BEI’ and the MOXO performance, within each group the MOXO performance appears to be related also to attention state. Previous studies have reported the state dynamics of CPT evaluations (Zabel et al., 2009). The limited within-group correlation between CPT and ASRS results (Vaughn et al., 2011) may suggest a more transient state-related effect on CPT results. Therefore, we suggest that CPT results and the 1-min auditory oddball BEI’ are sensitive to attention state within the groups of ADHD patients and controls, and that the 1-min BEI’ may be used for daily evaluation of attention, and possibly for titration of treatment (Gruber et al., 2007).

### Treatment Effect Captured by the Oddball BEI’

The functional improvement between the first and second MOXO CPTs may be ascribed to treatment, but also to learning or to accommodation with the task, as a function of time (Gualtieri and Johnson, 2005).The MOXO BEI’ improved with both treatment and time/learning effects. This is not surprising, because it is reasonable to assume that learning or task accommodation also increase attention within the task. At the same time, the oddball BEI’ improved for ADHD participants with the treatment effect, but not with the time/learning effect. This may be viewed as yet another evidence of the efficacy of the oddball BEI’ in monitoring the basic attention state, which is directly affected by pharmacological treatment. The sensitivity of the oddball BEI’ to treatment effect could serve as an effective tool for treatment titration, which may be valuable given the dynamics in response to treatment over time (Vitiello et al., 2001).

### Need for Further Research

The results of this pilot study suggest that BEI’ is a simple tool that may have important uses in diagnosis and treatment titration. But because this was a small-scale pilot study, larger, blinded studies are required to confirm the present findings.

## Author Contributions

GS is the developer of the electrophysiological marker. YB is the principal investigator (PI). UN, GE and SM recruited the patients and performed clinical evaluations.

## Conflict of Interest Statement

The research was funded by BrainMARC Ltd., the developer of the BEI (the electrphysiological index evaluated). GS is co-founder and chief scientist of BrainMARC Ltd. YB served as principal investigator in the study, which was conducted at his clinical center. The other authors declare that the research was conducted in the absence of any commercial or financial relationships that could be construed as a potential conflict of interest.
